# A hidden face of migration: Diabetic ketoacidosis in a severely malnourished refugee

**DOI:** 10.1002/ccr3.2508

**Published:** 2019-11-06

**Authors:** Suzanne Sap, Yanelle Wandji Nzebia, Viola Dohvoma Andin, Paule Yvette Hagbe, Ritha Mbono, Irene Fotang Tangmoh, Paul Olivier Koki

**Affiliations:** ^1^ Department of pediatric endocrinology, Mother and Child Center of Yaounde Yaounde Cameroon; ^2^ Department of ophthalmology Yaounde central hospital Yaounde Cameroon; ^3^ Pediatric Department Mother and Child Center of Yaounde Yaounde Cameroon

**Keywords:** ketoacidosis, migration, severe malnutrition, type I diabetes

## Abstract

Besides violence, the risk of under nutrition and infection, migrant children with noncommunicable chronic diseases face serious challenges in the management of their conditions. Management of diabetic ketoacidosis in a severely malnourished patient includes careful hydration, therapeutic feeding, and monitoring.

## Graphical abstract

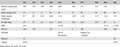

## 1 INTRODUCTION

We report the case of a 14‐year‐old refugee, presenting with severe acute on chronic malnutrition and diabetic ketoacidosis. This case highlights management difficulties and raises concerns on the management of diabetes in migrants (refugee and internally displaced people).

The increasing number of refugees and internally displaced people has a profound implication on health, with an outbreak of severe malnutrition and infections preventable by vaccination (eg, poliomyelitis and measles).^1^, ^2^ Children with diabetes and other noncommunicable diseases in this sanitary crisis are not seen as priorities by currents health policies.^3^ Policies to fight against disease outbreaks usually outweigh those to control noncommunicable diseases such as diabetes. We report the case of an adolescent girl presented with diabetic ketoacidosis and malnutrition, with the aim of raising awareness on the management challenges of diabetic ketoacidosis and acute on chronic malnutrition.

## 2 CASE REPORT

A 14‐year‐old girl, living in a refugee camp, was referred from a district hospital for the management of altered level of consciousness, fever, and hyperglycemia. Fever started 2 weeks prior to consultation for which she received empiric treatment for malaria in a district hospital. She was also diagnosed with severe acute on chronic malnutrition for which she received ready‐to‐use therapeutic food. The situation was worsened by polyuria, excessive thirst, uncontrolled hyperglycemia, persistent fever and progressive lethargy, necessitating her transfer to the endocrinology unit of the Mother and Child Center of Yaounde.

Past history was remarkable for diabetes which was diagnosed a year ago after 2 months of polyuria and excessive thirst. Insulin therapy was started at 0.5 IU/Kg/day with mixed insulin but patient's compliance was poor. She reported loss of sight 7 months ago (3 months after the diagnosis of diabetes). She is the 3rd child in a nonconsanguineous family of 10 children of whom three are alive. They fled the Central African Republic to Cameroon and for the past 4 years have been living in a refugee camp in the East region of Cameroon. Neither she nor the mother went to school.

On admission, she was lethargic (Glasgow coma scale was 13/15). Her weight was 19 kg (<3rd percentile), her height was 133 cm (<3rd percentile) for a BMI of 10.7 kg/m^2^, −5.5 SD for age. Vital signs were as follows: blood pressure: 100/80 mm Hg, pulse: 100 bpm, respiratory rate 34 cycles/min and temperature was 36.8°C. She had fine hair and poor dental hygiene. Her Tanner stage was B1P1. Her abdomen was distended with no palpable mass. She had vulvovaginal erythema. She had a dry mouth, sunken eyes, mixed signs of dehydration and severe malnutrition (skin pinch went back slowly) and bilateral leucocoria.

Her serum glucose level was 440 mg/dL (24.4 mmol/L), K + 3.3 mmol/L, corrected Na + 148.4 mmol/L, Cl‐108 mmol/L. HbA1C was 12% (108 mmol/mol). Urinalysis revealed nitrites (3+), ketones (2+), glycosuria (3+), and culture later on showed growth of *E coli*. Her hemoglobin level was 12.3 g/dL, white blood cell count was 9500/mm^3,^ and platelets count was 308 000/mm^3^. Aspartate aminotransferase (ASAT) and alanine aminotransferase (ALAT) were in the upper limits (42 and 44 IU/L, respectively) and renal function was normal (blood urea nitrogen (BUN) 0.07 g/L, serum creatinine 3 mg/L). Blood proteins were 93 g/L. Blood gases were not available.

She was admitted to the intensive care unit. She received oral rehydration solution for the malnourished (10 mL/kg/hr for 2 hours) followed by 1.5 L/m^2^/day of normal saline intravenously (IV) and KCL 1.5 g/L (IV) for 24 hours. Rapid‐acting insulin was started 2 hours later with an hourly subcutaneous dose of 0.1 IU/Kg. An antibiotic was also given (ceftriaxone at 50 mg/kg/day) as well as routine de‐worming with a single dose of 400 mg of albendazole. Four hours following admission, she was more reactive; ketonuria was 1+, serum glucose level was 408 mg/dL (22.4 mmol/L). Rapid‐acting insulin was replaced by multiple injections using neutral protamine Hagedorn insulin (NPH) and actrapid (1IU/kg/day). Ready‐to‐use therapeutic food F75 (75 kcal/100 mL) was started at 130 mL/kg/day divided into eight meals. Folic acid (5 mg once) and vitamin A (200 mg on days 1, 2, and 14) were added to the treatment.

The day after admission, she developed lower limb edema and abdominal distension without ascites and IV fluids were stopped. The oedema regressed on day 2. On day 3, the F75 was replaced with F100 (100 kcal/100 mL) 130 ml/kg/day and progressive introduction of Plumpy Nut^®^ and normal food. Progression of her nutritional status is shown in Table 1.

**Table 1:** Evolution of the patient in wk

	W1	W2	W3	W4	W5	W6	W7	W8	W9	M5
Means of glycemia mg/L	188	167	180	290	260	140	151	147	138	?
Daily insulin (UI)	14	11	11	16	28	32	34	36	36	36
Daily insulin (UI/Kg/d)	0.7	0.5	0.47	0.68	1.14	1.25	1.28	1.27	1.24	1.15
Weight (Kg)	19	22	23	23.5	24.4	25.6	26.5	28.3	29	32
BMI	10.7				13.28				15.35	17.56
MUAC	130	136		155		0	160		160	160
Incident					Fever, Bronchitis		Surgery for cataract			
HbA1C	12									10
Tanner	S1P1									S1P1

Abbreviations: M, month, W, weeks.

Communication with and education of patient and her family was difficult because of unavailability of a translator trained in medical terminology and the fact that the patient was almost blind. This was partially improved following her cataract surgery performed 2 months later.

Upon discharge after 9 weeks spent in hospital, her weight was 29 kg, height 133 cm (BMI 16.4 kg/m^2^) and MUAC of 160 mm. She was discharged to her refugee camp on multiple insulin injections. Two months later, her weight was 32 kg, height 135 mm (BMI 17.56 kg/m^2^) and HbA1 C 10%.

## 3 DISCUSSION

Insecurity is known to severely affect healthcare systems especially in low‐income countries, reducing even the minimal care services available.^1^, ^2^, ^3^ Because of the political crisis in the Central African Republic, 460 000 people are displaced to neighboring countries including Cameroon (East Region).^1^ A similar situation is seen in the northern part of Cameroon, where 6% of children living with diabetes were internally displaced and that insecurity was an indirect cause of uncontrolled glycaemic level. 4

One of the major consequences of insecurity is food insufficiency.^1^, ^2^, ^3^ It is known that insulin insufficiency leads to nutritional deficiency known as Mauriac syndrome in type I diabetes. 5 But in the present case, severe malnutrition (acute and chronic) was probably multifactorial and not exclusively explained by insulin insufficiency. Yet, severe stunting was suggestive of a prolonged insufficient nutritional intake recently worsened by insulin insufficiency. Nonetheless, no hepatomegaly was found in the girl, which is not classical of a Mauriac syndrome. This also suggests an acute component of under nutrition, presenting as severe wasting found (BMI −5.5 SD).^5^ In fact, chronic food insufficiency is known to lead to a reduction of beta cell mass^6^, ^7^ which might be an accelerating factor of developing diabetes in this patient. The girl also showed clinical signs of micronutrient deficiency, systematically corrected accordingly to WHO.^8^ Prolonged insecurity, food inadequacy, and unavailability are known to be the foundation of malnutrition in low‐income countries. 1, 2, 3, 7 The latter is the source of high mortality rate in children in these contexts. Therefore, modification of social conditions of patients living with diabetes is a big challenge, even though insulin is available to ensure their survival. These social conditions can be improved by community interventions such as literacy, patient education in local languages, sustainable micro‐projects for families to increase food access.

On admission, the patient presented with two life‐threatening conditions: ketoacidosis (DKA) and acute severe malnutrition. These conditions have contradictory approach in management: the first requires rehydration and the 2nd requires controlled fluid restriction.^8^

Correction of fluids loss started with oral rehydration solution for malnourished. We put the lowest quantity of IV fluids recommended for DKA management (1.5 L/m^2^)^9^ but this lead to oedema the day after. Fluids overload is a dreaded complication in severe malnutrition.

If in Mauriac syndrome appropriate insulin delivery improves nutritional status,^5^ the present case also required nutritional rehabilitation. The latter was based on multiple meals 6 providing 75 kcal/100mL, followed by transitional food (100 kcal/100mL). This leads to another dilemma, how to monitor this nutritional rehabilitation in the absence of continuous glucose monitoring and continuous insulin delivery. This topic remained unanswered in the reviewed literature.

We observed a slow decrease decline of serum glucose level within the first 24 hours and no acute complication of initial treatment of severe malnutrition (hypoglycemia). We considered oedema observed as a sign of fluid overload and not re feeding syndrome as the patient had insulin deficiency.^10^

During the transitional phase, she showed high insulin requirements for glycemic control. This was explained by high amount of carbohydrates contained in therapeutic food, given at a high frequency. High insulin requirement may also suggest a certain level of peripheral insulin resistance described in malnutrition.^11^ Unsurprisingly, the patient had a pubertal delay, consequence of chronic food insufficiency. She also presented with bilateral cataract.

Another major dilemma raised by the present case is patient education. Neither the mother nor the child have ever learnt to read or write and were expressing themselves in a language uncommon in our setting. Communication required translators who had never heard about diabetes. The final information received by patient and mother was sometimes incorrect, explaining partly the prolonged hospitalization.

Despite a slight improvement of nutritional status after 2 months spent in the camp, glycemic control was not effective.

## 4 CONCLUSION

The present case emphasizes a number of management challenges seen in low‐income settings, worsened by insecurity, food unavailability, and illiteracy. Although insulin is available in our country thanks to the CDIC program, a more holistic educational program is urgently needed for health personnel in charge of refugees, children, and families. In addition, advocacy dedicated to noncommunicable diseases is essential for children living in unstable areas.

## CONFLICT OF INTEREST

The authors declare that there is no conflict of interest regarding the publication of this article.

## AUTHOR CONTRIBUTION

All authors contributed to the care of this patient. VAD: managed the bilateral cataract of the patient. All authors critically revised the manuscript. SS and YWN: were responsible for the preparation of the manuscript.
